# Differential impact of the school context on ethnic and racial identity and depression for monoracial and multiracial early adolescents

**DOI:** 10.3389/fpsyt.2023.1080085

**Published:** 2023-11-17

**Authors:** Cindy Y. Huang, Emily Hunt, Elizabeth A. Stormshak

**Affiliations:** ^1^Department of Counseling and Clinical Psychology, Teachers College, Columbia University, New York, NY, United States; ^2^Counseling Psychology and Human Services, College of Education, University of Oregon, Eugene, OR, United States

**Keywords:** ethnic and racial identity, racial minority adolescents, multiracial adolescents, school context, depression

## Abstract

**Introduction:**

This study examined the direct and indirect effects of school context (negative peer relationships, school environment) on ethnic and racial identity (ERI) development in middle school and later depression symptoms in high school. Differences by racial group were examined for non-Hispanic White (NHW) early adolescents, monoracial adolescents, and multiracial adolescents.

**Methods:**

This study used existing data from a large, multiwave, longitudinal study that included 593 racial/ethnically diverse adolescents from sixth grade through ninth grade across three public middle schools in the Pacific Northwest.

**Results:**

Using multigroup path analysis in structural equation modeling, the findings indicated differences by racial group—school environment was associated with positive ERI development in middle school for NHW and monoracial adolescents but not for multiracial adolescents. For multiracial adolescents, ERI predicted later depression symptoms.

**Discussion:**

These findings demonstrated the importance of examining school context and peer relationships in relation to ERI development and psychological wellbeing.

## Introduction

The youth population of the United States (U.S.) has officially achieved a “majority-minority” status, meaning the majority of children under 15 are a racial/ethnic minority (1). The most recent Census revealed that 60.4% of U.S. youth are now comprised of individuals who identify as Black, Asian, Hispanic, and multiracial (2). Additionally, the multiracial population alone has significantly increased since 2000—an estimated 32% of young people under 18 years belong to two or more racial groups (3). The diverse racial makeup of the American people suggests that the majority of youth in the U.S. are developing as either monoracial or multiracial minorities. While researchers have advanced the knowledge base of racial/ethnic minority youth development over the past few decades, the majority of this work have focused primarily on monoracial individuals. It is important to examine the differences and similarities across monoracial and multiracial adolescents in order to better understand the complexities of racial/ethnic minority youth development.

One crucial aspect of racial/ethnic minority youth development is ethnic and racial identity (ERI). ERI is defined as a multidimensional psychological construct that captures the beliefs and attitudes that individual have about their ethnic-racial group membership, and the processes that leads to the development of these beliefs and attitudes (4). Higher levels of ERI are associated with a variety of positive youth outcomes, such as self-esteem, academic achievement, and less substance use (5–9). Higher levels of ERI are also associated with positive psychological adjustment including fewer depressive symptoms and aggressive behaviors (5–7). Developed by the Ethnic and Racial Identity in the 21st Century Study Group (4), the concept of ERI emerged as a way to better capture the extant literature on racial identity and ethnic identity. The Study Group—comprised of scholars in the field of racial/ethnic minority youth development—recommended against distinguishing between racial identity and ethnic identity, given the overlap between the two constructs. For example, studies have found that children develop their awareness of ethnicity and race through similar processes, and follow similar trajectories of growth (8–10). Parents frequently socialize (i.e., teach) their children about their racial and ethnic group membership by combining race and ethnicity (11). Research has also found that the processes through which ethnic identity develops are typically racial in nature. For instance, experiences were racial discrimination was associated with increasing exploration of ethnic identity among Black adolescents (9). Conversely, ethnic identity was found to be associated with racial identity beliefs (10). This study examined the ERI of early adolescents by examining ethnic identity. Specifically, the study examined the influence of the school context – defined by peer relationships and the school environment—and its impact on ERI in middle school.

### Early adolescence and ERI

Early adolescence is a time when youth begin to experience changes in ERI (12, 13). Studies have found that levels of ethnic affirmation increased during the transition into middle school for adolescents, especially African American and Latino youth (13). Other studies examining the longitudinal growth of ethnic identity have found that the majority of youth remain stable or increase in ERI levels over early and middle adolescence school (14–16). Research suggests these changes are influenced by the growing salience of ethnicity (16), highlighted by experiences and relationships in the school environment (17). In fact, studies have found a tendency for middle school youth to segregate themselves by their race and ethnicity compared to elementary school (18, 19). Thus, the emerging salience of ethnicity, along with the biological, psychological, and social changes during early adolescence, underscores the importance of examining ERI and its impact on adolescent wellbeing in this critical developmental period.

### The school context and ERI for monoracial and multiracial minority youth

The extant literature suggests that racial/ethnic minority youth frequently have negative experiences with their peers at school. Studies have found that racial/ethnic minority students reported more perceived victimization than their non-Hispanic White (NHW) peers (20), specifically due to their ethnicity (21, 22). Research further demonstrates that African American and Hispanic adolescents report high rates of victimization from peers—87% of African American adolescents and 76% of Hispanic adolescents endorsing at least one discriminatory experience from their peers (23, 24). The differential treatment of youth based on their ethnicity exists in the school environment—as many as 46% of African American and 50% of Hispanic/Latino students have reported receiving lower grades based on their race/ethnicity (25), and disparities in the number of office referrals and suspensions for African American and Hispanic youth are well documented (26, 27). Furthermore, teachers have higher behavioral expectations of NHW youth than of minority youth (28). Racial/ethnic minority youth also perceive differing levels of support from their teachers based on their race and ethnicity, with NHW students reporting higher levels of perceived support from teachers than Chinese American and African American students (29, 30).

These findings are concerning, as negative peer and school experiences have detrimental effects on the identity development of racial/ethnic minority youth. Researchers have found that peer rejection and harassment based on racial/ethnic background are associated with negative ethnic identity beliefs (31, 32). Discrimination from peers is also associated with adolescents’ perceptions of their own ethnic group (25). Moreover, negative experiences in the school environment are associated with higher levels of psychological distress. For instance, adolescents who report high levels of peer victimization are at higher risk for depression, anxiety, and behavioral and adjustment problems (33–35). Student perceptions of low teacher support are also associated with more depressive symptoms for youth (36–38). Given the prevalence of these experiences, racial/ethnic minority youth are at increased risk for negative mental health outcomes compared to their NHW peers.

The overwhelming majority of this research has been conducted on monoracial minority youth. As such, the existing knowledge base inadvertently surmises that multiracial youth have similar ERI processes, and have similar outcomes as monoracial adolescents. While research with biracial adults suggest higher ERI is associated with better psychological outcomes (39), emerging research is demonstrating that developmental trajectories and outcomes may differ for multiracial youth. Multiracial adolescents reported higher rates of substance use and aggressive behaviors, and are at a higher risk for general health problems, academic concerns, and engaging in negative activities (40–42). In addition, studies have found that multiracial adolescents have higher rates of substance use in adulthood and are a higher risk for high lifetime substance use compared to their mono-racial/ethnic peers (41, 43). Multiracial youth were found to be more aware of race/ethnicity-related issues at an earlier age than monoracial youth, likely due to the awareness of these issues by the people with whom they interact and the potential feelings of marginality as a result of these (40). A study found that the majority of multiracial adolescents experienced a moderate decline in their ERI level over the early adolescent years, while the majority of minority adolescents experienced an increase (15). Furthermore, a strong sense of ERI was found to actually increase the likelihood of engaging in aggressive behaviors for multiracial adolescents instead of serving as a protective factor (40).

For multiracial individuals, developing ERI can be an especially complex and challenging process because they must navigate the integration of two or more racial backgrounds (44). Multiracial youth may experience more difficulties in their identity development due to various factors (45). For example, they may not receive the support they need to develop an achieved identity because their parents, who typically identify with one single racial/ethnic group may not have the necessary awareness and/or willingness to talk about issues related to their children’s ethnic identities (45). One study found that the majority of multiracial adolescents reported not talking about race at home (46). Studies on multiracial adults have found they often identity denial, which is defined as experiences in which one’s biracial identity is denied or questioned by others (47). Multiracial individuals often feel forced to choose just one racial group to identify with due to ways that they are being perceived by others based on physical appearance, social class, and other cultural and contextual factors (44). They also can experience marginalization and rejection from not just one side of their racial/ethnic background but from both sides, not belonging to or being fully accepted by either group. These findings are not generally relevant or encountered by monoracial minority youth, and suggest that the influences on ERI development for multiracial youth may differ for monoracial youth.

### The current study

A surprisingly small number of studies have investigated the impact of the larger school context on ERI development and later psychological outcomes. Even fewer have examined how these relationships may differ for monoracial versus multiracial adolescents. This study examined the associations between negative peer experiences in schools, school environment, ethnic identity, and depression in a large sample of racial/ethnic minority early adolescents. Negative peer experiences in sixth grade was hypothesized to be associated with lower levels of ERI in middle school and higher levels of depression in ninth grade. Higher levels of ERI were hypothesized to be associated with lower levels of depression in ninth grade. ERI was hypothesized as a mediator in the relationship between negative peer experiences and school environment in sixth grade and depression in ninth grade.

## Methods

This study used existing data from a large, multi-wave, longitudinal study examining the psychological, social, and behavioral development of adolescents starting in middle school. Sixth grade adolescents and their parents were recruited from three public middle schools in an urban area of the Pacific Northwest. Consent forms were mailed or sent home to parents; all parents of sixth grade students were invited to participate in the study. Approximately 80% of all parents agreed to participate, resulting in 593 enrolled adolescents. Students enrolled in the study were provided with self-report surveys that were completed once a year during the school year. Students completed surveys in sixth grade (Time 1), seventh grade (Time 2), eighth grade (Time 3), and ninth grade (Time 4). The full sample comprised 51% male participants and 49% female participants, and the racial/ethnic composition was as follows: European American, 36%; Latino/Hispanic, 18%; African American, 15%; Asian/Pacific Islander, 9%; American Indian, 2%; and biracial/mixed ethnicity, 19%. More than 80% of youth were retained across the 4 years of the study (48).

### Participants

The 593 adolescents comprised of 51.5% males (*n* = 305) and 48.5% females (*n* = 287). The mean age at sixth grade was 11.90 years, and the ethnic composition was as follows: 35.9% NHW adolescents (*n* = 213), 18% Latino adolescents (*n* = 107), 15.2% African American adolescents (*n* = 90), 8.9% Asian/Pacific Islander American (*n* = 53), 2.5% American Indian/Native American (*n* = 15), and 19.2% multiracial adolescents (*n* = 114). In order to address the research questions for the present study, participants who self-identified as a person of color with one race (e.g., only African American, only Latino) were combined into one group labeled *monoracial* to allow for comparison with multiracial adolescents. Monoracial adolescents represented 44.5% (*n* = 264) of the adolescents in this study.

### Measures

All study variables were measured using the Child and Family Center Youth Questionnaire (49, 50), which included a range of items that assess for adolescent social, emotional, and behavioral wellbeing.

#### Demographic information

Adolescents self-reported their age, gender, and race/ethnicity.

#### Ethnic racial identity

An eight-item short version of the Multigroup Ethnic Identity Measure (MEIM) (51) was used to obtain adolescent self-report of levels of ERI. Adolescents were instructed to think of an ethnic group they felt they belonged to, and reference that group as they responded to the measure items. The MEIM has been shown to be reliable for diverse groups of adolescents (*α* = 0.81) (7). Items are rated on a 4-point scale ranging from 1 (*strongly disagree*) to 4 (*strongly agree*) and include statements such as “I know what being in my ethnic group means to me” and “I feel good about my cultural or ethnic background.” The ethnic identity mean score is obtained by summing across all the items in the measure. This abbreviated version of the MEIM has been shown to be reliable (14). In this study, ethnic identity was measured from Grades 6 through 9 (Waves 1–4) and showed high reliability through all waves (*α*s = 0.90, 0.90, 0.93, 0.92, respectively).

### Negative peer relationships

The latent construct of negative peer relationships was measured using three measures drawn from the larger Child and Family Youth Questionnaire, which assessed for youth’s peer and social skills (50). These questions have been widely validated in studies with diverse populations of youth (52, 53). *Perception of peers* is a seven-item youth self-report measure was used to assess adolescents’ perceptions of the peers at their school. Each item ranged on a scale from 1 to 5 and had a descriptive word used to describe the peers. Items were *unfair–fair*, *mean–nice*, *cold–warm*, *unfriendly–friendly, bad–good*, *cruel–kind,* and *dishonest–honest*, and higher scores indicated more-favorable perceptions of other students. Wave 1 (Grade 6) of this measure was included as part of the latent variable for negative peer relationships. This measure was reliable (*α* = 0.91). *Problems with peers* was assessed using this four-item measure on the frequency of problems that adolescents had with other students in the past month. On a 5-point scale ranging from *never or almost never* to *always or almost always,* youth rated their level of agreement to questions such as “I had a problem with other students,” “Students called me names, swore at me, or said mean things to me,” and “A student hit, pushed, or fought me.” Wave 1 was included in the analysis and partially comprised the latent variable of negative peer relationships. Reliability of the measure was *α* = 0.77 at Wave 1. *Teased by peers* was assessed using a five-item measure that determined the frequency of teasing experienced by youth at their school. On a scale of 1–5, adolescents rated their level of agreement ranging from *never or almost never* to *always or almost always* on items such as “I was teased by kids at school for no reason” and “I was ignored by kids I wanted to hang out with.” The measure in sixth grade (Wave 1) was included as the latent variable measuring negative peer relationships. Reliability of this measure was *α* = 0.78 at Wave 1.

### School environment

The latent construct of school environment was measured using three measures assessing youth perception of their teachers, their school climate, and school safety. *Perception of teachers* included seven items and asked about adolescents’ perceptions of their teachers at school. Youth used a scale of 1–5 to assess the following qualities: *unfair–fair, mean–nice, cold–warm, unfriendly–friendly, bad–good, cruel–kind,* and *dishonest–honest,* with higher scores indicating more-favorable perceptions of teachers. This measure was reliable in this sample (*α* = 0.91). *School climate* was a nine-item measure that evaluated adolescents’ positive experiences at school, including availability of teachers, amount of praise received, and opportunities to be involved in extracurricular activities. Youth rated their experiences on a 5-point Likert scale ranging from *never to almost never* to *always or almost always.* The reliability of the measure at Wave 1 was 0.73. *School safety perception* asked youth to report how safe they perceived their school to be in sixth grade (Wave 1) was used as part of the school environment latent variable. Adolescents rated their level of agreement from *strongly agree* to *strongly disagree* on seven items, such as “I feel safe in the school hallways,” “I feel safe in the school restroom,” “I feel safe in the classroom,” and “I feel safe outside the school.” This measure was reliable (*α* = 0.88).

#### Depression symptoms

Youth completed a 14-item assessment as a part of the larger survey used in previous research to assess diagnostic symptoms of depression (50). Adolescents reported on items that described their feelings and ideas in the past month, such as feeling sad or depressed, cranky or grumpy, or having sleep problems. Items were measured on a 5-point Likert scale ranging from 1 (*never or almost never*) to 5 (*always or almost always*). Wave 1 of this measure was used as a control variable and Wave 4 depression (ninth grade) was the outcome. Both waves were found to be reliable (*α*s = 0.95).

### Data analysis

**Figure 1:**
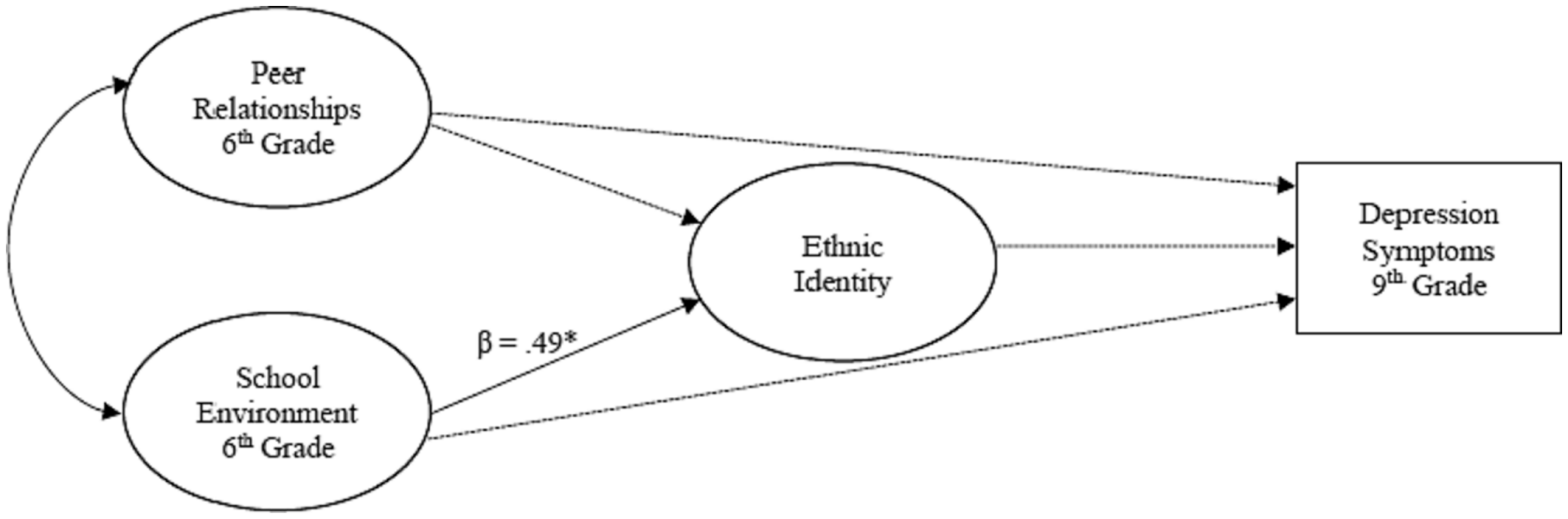
The influence of the school context on ethnic identity development and later depression symptoms for Non-Hispanic White (NHW) adolescents. ***p < .001, **p < .01. *p < .05.

**Figure 2:**
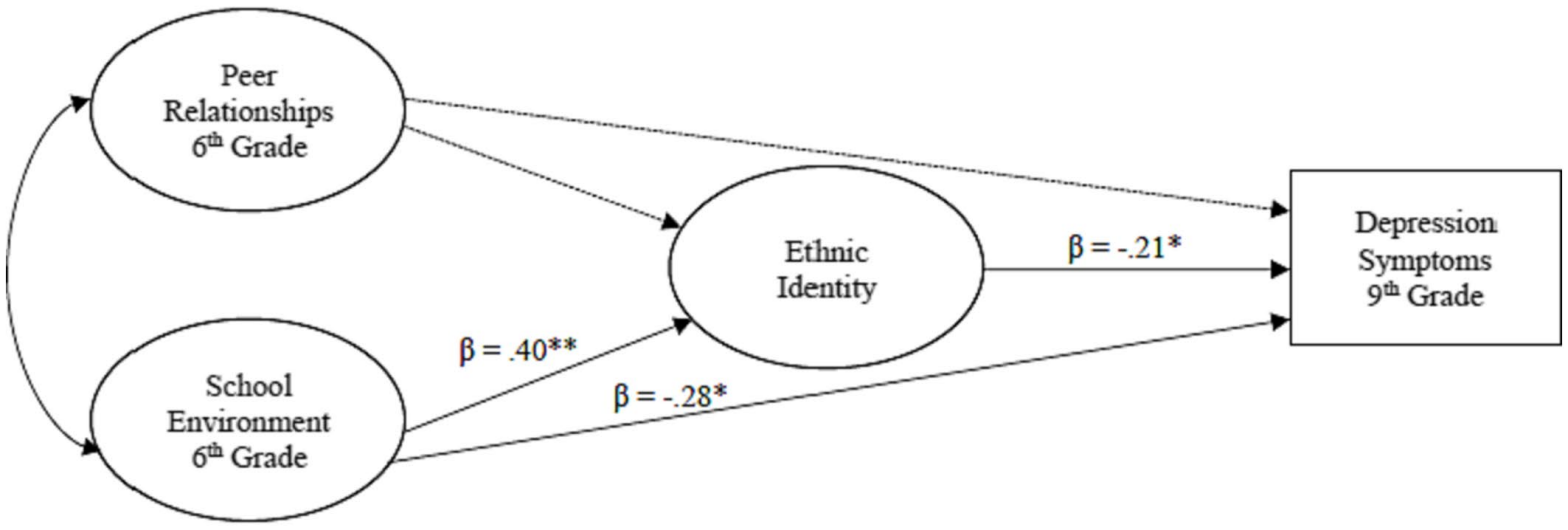
The influence of the school context on ethnic identity development and later depression symptoms for monoracial adolescents.***p < .001, **p < .01. *p < .05.

**Figure 3:**
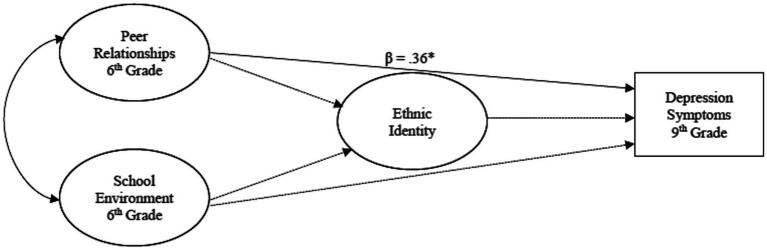
The influence of the school context on ethnic identity development and later depression symptoms for multiracial adolescents. ***p < .001, **p < .01. *p < .05.

A multigroup, path analysis was conducted using structural equation modeling (SEM) to determine the mediational role of youth ethnic identity in the relationship among negative peer relationships and the school environment in middle school and their impact on depression symptoms in ninth grade (see Figures 1–3). Direct and indirect effects were examined. Adolescent gender and baseline depression symptoms were covariates in the analyses. Multigroup analysis (MGA) was used to determine if these relationships differed by racial group (NHW, monoracial, multiracial). All analyses were conducted using AMOS software version 28. Missing data was imputed using multiple imputation with the Bayesian approach (54). This approach has been found to achieve better and more reliable results (55). The following indices were used to assess model fit: comparative fit index (CFI) (56), with values greater than 0.95 indicating good model fit and root mean square error of approximation (RMSEA) (56), with values less than 0.08 indicating reasonable model fit. A model was determined to fit well if these criteria were met. A statistical significance level of 0.05 was used to evaluate the statistical significance of individual model parameters (e.g., factor loadings, correlations).

Racial group differences were determined using MGA (57). First, an unconditional model was examined, which allowed for all parameters to be freely estimated across groups. Next, a constrained model was examined—this model constrained all parameters at the measurement level (e.g., path coefficients) to be equal across racial groups. The chi-square difference test was used to determine if the unconstrained and constrained models differed significantly – non-significance indicates no differences in parameters between groups, whereas significance indicates a lack of equality in the constrained model and differences across groups.

## Results

Descriptive statistics for all variables were examined, including mean, standard deviation, and frequency distributions, to examine the tenability of assumptions required for the proposed statistical analyses. Correlations between independent variables were evaluated with a bivariate correlation matrix and found to be small to moderate, providing evidence that multicollinearity was not a problem. Extreme skew and kurtosis values were examined. The majority of study variables was found to be within the recommended limits of ±3.0 to 3.0 for skew values, and −10.0 to +10.0 for kurtosis values (58), with the exception of youth experiences of discrimination at Waves 1, 3, and 4 (skews = 3.61, 3.03, and 3.30, respectively; kurtosis = 17.40, 12.04, and 13.90, respectively). Despite this, the use of Bayesian estimation is robust and can accurately estimate the model despite a non-normal distribution (58).

### School context, ethnic identity, and later depression symptoms

The model demonstrated relatively good fit, χ^2^(30) = 114.37, *p* < 0.001, CFI = 0.92, RMSEA = 0.07, SRMR = 0.07. To determine if the model path coefficients differed by racial group, the chi-square difference between the measurement and unconditional models were examined – this test was statistically significant (*p* < 0.05), indicating the path coefficients in the model were not equal across racial groups. Thus, the direct and indirect paths were examined by racial group and discussed separately.

#### NHW adolescents

A significant and positive direct effect was found for the relationship between school environment and ERI in middle school (β = 0.49, *p* < 0.05); a positive school environment was associated with positive changes in ERI. No additional direct or indirect effects were found for NHW (see Table 1). Figure 1 depicts the conceptual model for these adolescents.

**Table 1:** Direct and indirect effects for NHW, monoracial, and multiracial adolescents.

	Direct effects		Indirect effects
	Ethnic identity	Depression	Depression
NHW adolescents (*n* = 213)			
Negative peer relationships	0.63	−0.47	0.17
School environment	1.07*	0.57	−0.28
Monoracial adolescents (*n* = 264)			
Negative peer relationships	−0.35	0.21	0.03
School environment	0.76**	−0.45	−0.06
Multiracial adolescents (*n* = 114)			
Negative peer relationships	−0.24	0.47**	0.01
School environment	−0.16	0.34	0.004

***p < 0.001, **p < 0.01, *p < 0.05.

#### Monoracial adolescents

As shown in Figure 2, school environment in sixth grade was significantly and positively related to ERI in middle school, *β* = 0.40, *p* < 0.01. School environment was also found to be significantly and negatively related to later depression symptoms, *β* = −0.28, *p* < 0.05, suggesting that poorer perceptions of the school environment was associated with higher levels of depression symptoms in ninth grade. ERI in middle school was negatively associated later depression symptoms for monoracial adolescents, *β* = −0.21, *p* < 0.05—the more negative the peer relationships in sixth grade, the higher the depression symptoms in ninth grade. Peer relationships were not significantly associated with ERI or depression symptoms (*p*s > 0.05), and no indirect effects were found.

#### Multiracial adolescents

No direct effects were found for the relationships from the predictors to ERI in middle school; however, negative peer relationships in sixth grade were positively associated with later depression symptoms, *β* = 0.36, *p* = 0.05, indicating that higher levels of negative peer relationships in sixth grade was associated with more depression symptoms in ninth grade for these adolescents. No significant indirect effects were found for the multiracial adolescents (see Figure 3).

## Discussion

This study examined how the school context in early middle school (sixth grade) may impact ERI through early adolescence and depression symptoms in high school (9th grade). In the unconditional model, peer relationships affected adolescent ethnic identity development over early adolescence. Specifically, adolescents who reported higher levels of negative peer relationships in sixth grade had lower levels of ERI. Higher levels of ERI were associated with lower levels of depression in ninth grade. This makes sense, because youth with more positive peer interactions are more likely to experience their school environment as positive and feel more connected to their schools, and therefore are more likely to experience positive outcomes (37, 38). Existing research supports this finding; adolescents who perceive a positive school climate have better academic and psychosocial outcomes, such as decreased depression (38).

No indirect effects were found, suggesting that ERI did not play a role in the relationship between peer relationships, school environment, and later depression symptoms. This is indicative of the powerful influence of peers at this age, and ERI may also not be salient enough for early adolescents to be an effective buffer against discrimination, teasing, and bullying. This further highlights the need for additional research and improved understanding of how school context and other school contextual factors (e.g., discrimination) may influence psychological outcomes for early adolescents.

Exploration of differences between racial groups yielded interesting results. This model yielded the most significant relationships for monoracial adolescents, supporting the hypothesis that developmental trajectories for multiracial adolescents may differ from those of NHW adolescents and those of monoracial adolescents. Only one significant direct effect was found for NHW adolescents—school environment was related to ERI for NHW adolescents; specifically, a positive school environment was related to greater ERI. Interestingly, NHW are often not included in studies examining adolescent ERI; it has been argued that ERI is less salient and prominent for these youth than it is for minority youth (59). Thus, this finding suggests NHW adolescents may be more aware of their ERI during early adolescence than was previously expected. A study examining youth awareness of bias found that by middle school, NHW adolescents were as equally aware of ethnic bias as were African American and Latino youth (60). Moreover, studies have suggested that in diverse communities, race and ethnicity may be just as salient for majority youth as it is for minority youth (61). This is especially true when a large portion of the youth of NHW descent identify as having come from a cultural group that has experiences with discrimination (e.g., Jews).

For monoracial adolescents, the school environment significantly predicted ERI and later depression symptoms. More specifically, a positive school environment in sixth grade was associated with higher ethnic identity levels and with lower levels of depression symptoms in ninth grade. ERI was also found to predict later depression symptoms. These findings support existing research that ERI is a critical component of one’s identity and is associated with psychological wellbeing for racial/ethnic minority adolescents. These finding suggest that ethnic identity can potentially act as a protective factor in a negative school context, emphasizing the need to facilitate the development of ERI for adolescents of color.

For multiracial youth, peer relationships were found to be a significant predictor of later depression symptoms. Specifically, multiracial youth who reported more negative peer relationships experienced more depression symptoms in high school. Given that school environment was not significantly associated with any variables, it can be deduced that peers play a particularly influential role in the school experiences of multiracial adolescents, especially because this finding was significant only for this group. For example, peers may provide additional support for these youth during early adolescence, a period of increasing identity exploration; in fact, multiracial youth experience more ERI exploration and affirmation within the context of schools than their monoracial NHW peers (62). This can be especially true considering the theory that multiracial youth may not receive sufficient support from their parents and caregivers with respect to identity development (7), which in turn drives adolescents to seek support in their social context. Research on the friendships of multiracial adolescents has found that the majority of multiracial adolescents who were more strongly aligned with just one of their racial identities (e.g., just Black or just NHW instead of Black and NHW) had more monoracial peers (e.g., only Black or only NHW friends), whereas adolescents who had more-integrated identities had a mix of monoracial and multiracial friends (63). Because the ERI development of multiracial adolescents is complex, it is plausible that these youth seek peer relationships that help them explore and express their identity.

## Conclusion and limitations

School context, defined by peer interactions at school, school climate, teacher support, and school safety, plays a significant role in youth development during early adolescence. Our study results indicated that school environment is particularly significant for monoracial adolescents (i.e., NHW, monoracial minority youth), and peer relationships are especially relevant for multiracial adolescents’ psychological wellbeing. This study contributes to the large existing body of literature about ERI, confirming the significance of a healthy ERI for adolescent development and mental health outcomes. It also provides much-needed knowledge of how social ecologies other than family characteristics contribute to ERI development.

This study had limitations that warrant mention. Although the study sample was diverse, the adolescent participants reside in the Pacific Northwest, a region that may not reflect the same diversity of larger metropolitan areas in the United States, such as Los Angeles or New York City. As such, the racial/ethnic diversity of this region should be considered while interpreting the study results, because results may differ with a sample from another region in the country. Other researchers should consider replicating and exploring similar research questions in cities with more racial/ethnic diversity. Another key limitation of the study was the use of only youth report on the study variables. Subsequent studies could build upon these findings by including additional measures of school context, such as teacher report of teacher–student relationships. Direct observation of school variables (e.g., peer and teacher interactions, acts of discrimination, school safety) would also provide a wealth of information to other researchers interested in capturing the optimal portrayal of peer relationships and the overall school environment.

In conclusion, implications of this study include enhanced understanding of how school context influences adolescent ERI in middle school and later depression symptoms. More specifically, understanding how, and to what extent, ethnic identity is influenced by school factors can enable prevention scientists to develop and incorporate relevant components into preexisting schoolwide interventions (e.g., Positive Behavior Support) (64) to help diverse adolescents form a healthy identity. Examples include promoting cultural sensitivity among all students and staff, decreasing bullying and other negative social peer interactions, and improving the overall school environment. Promotion of these positive attributes could help decrease the risk of poor academic and psychological outcomes for adolescents at risk of experiencing adversity.

## Data availability statement

The raw data supporting the conclusions of this article will be made available by the authors, without undue reservation.

## Ethics statement

The studies involving humans were approved by University of Oregon Institutional Review Board. The studies were conducted in accordance with the local legislation and institutional requirements. Written informed consent for participation in this study was provided by the participants’ legal guardians/next of kin.

## Author contributions

CH designed the study, carried out the data analysis, and wrote the manuscript. EH supported in the manuscript writing and preparations for submission. ES was the primary investigator of the study from which the data in this study originated, and contributed to the interpretation of the results. All authors provided feedback and helped shape the research and manuscript.

## References

[1] Fey W. (2020). The nation is diversifying even faster than predicted, according to new census data. Available at: https://www.brookings.edu/research/new-census-data-shows-the-nation-is-diversifying-even-faster-than-predicted/

[2] United States Bureau of the Census. National population by characteristics: 2010–2019. Washington, DC: U. S. Census Bureau (2019) Available at: https://www.census.gov/data/tables/time-series/demo/popest/2010s-national-detail.html.

[3] Rico B. Jacobs P. Coritz A. (2023). 2020 census shows increase in multiracial population in all age categories. Census.gov. Available at: https://www.census.gov/library/stories/2023/06/nearly-a-third-reporting-two-or-more-races-under-18-in-2020.html

[4] Umaña-Taylor AJ Quintana SM Lee RM Cross WE Rivas-Drake D Schwartz SJ. Ethnic and racial identity revisited: an integrated conceptualization. Child Dev. (2014) 85:21–39. doi: 10.1111/cdev.12196, PMID: 24490890 PMC6673642

[5] Roberts R Phinney JS Masse L Chen Y Roberts C Romero A. The structure of ethnic identity in young adolescents from diverse ethnic minority groups. J Early Adolesc. (1999) 19:301–22. doi: 10.1177/0272431699019003001

[6] Rumbaugh NHWsell N Mitchell CM Spicer P The Voices of Indian Teens Project Team. A longitudinal study of self-esteem, cultural identity, and academic success among American Indian adolescents. Cult Divers Ethn Minor Psychol. (2009) 15:38–50. doi: 10.1037/a0013456, PMID: 19209979 PMC2678750

[7] Smith CO Levine DW Smith EP Dumas J Prinz RJ. A developmental perspective of the relationship of racial–ethnic identity to self-construct, achievement, and behavior in African American children. Cult Divers Ethn Minor Psychol. (2009) 15:145–57. doi: 10.1037/a0015538 19364201

[8] Quintana SM. Racial and ethnic identity: developmental perspectives and research. J Couns Psychol. (2007) 54:259–70. doi: 10.1037/0022-0167.54.3.259

[9] Pahl K Way N. Longitudinal trajectories of ethnic identity among urban black and Latino adolescents. Child Dev. (2006) 77:1403–15. doi: 10.1111/j.1467-8624.2006.00943.x, PMID: 16999807

[10] Cokley KO. Racial(ized) identity, ethnic identity, and Afrocentric values: conceptual and methodological challenges in understanding African American identity. J Couns Psychol. (2005) 52:517–26. doi: 10.1037/0022-0167.52.4.517

[11] Hughes D Rodriguez J Smith EP Johnson DJ Stevenson HC Spicer P. Parents' ethnic-racial socialization practices: a review of research and directions for future study. Dev Psychol. (2006) 42:747–70. doi: 10.1037/0012-1649.42.5.747 16953684

[12] Smith TB Silva L. Ethnic identity and personal well-being of people of color: a meta-analysis. J Couns Psychol. (2011) 58:42–60. doi: 10.1037/a0021528, PMID: 21171745

[13] French SE Seidman E Allen L Aber JL. The development of ethnic identity during adolescence. Dev Psychol. (2006) 42:1–10. doi: 10.1037/0012-1649.42.1.1 16420114

[14] Phinney JS Chavira V. Ethnic identity and self-esteem: an exploratory longitudinal study. J Adolesc. (1992) 15:271–81. doi: 10.1016/0140-1971(92)90030-9, PMID: 1447413

[15] Huang CY Stormshak EA. A longitudinal examination of early adolescence ethnic identity trajectories. Cult Divers Ethn Minor Psychol. (2011) 17:261–70. doi: 10.1037/a0023882, PMID: 21787058 PMC3144497

[16] Phinney JS. Stages of ethnic identity development in minority group adolescents. J Early Adolesc. (1989) 9:34–49. doi: 10.1177/0272431689091004

[17] Mandara J Gaylord-Harden N Richards MH Ragsdale BL. The effects of changes in racial identity and self-esteem on changes in African American adolescents’ mental health. Child Dev. (2009) 80:1660–75. doi: 10.1111/j.1467-8624.2009.01360.x, PMID: 19930344

[18] Seidman E Aber JL French SE. The organization of schooling and adolescent development In: Maton KI Schellenbach CJ Leadbeater BJ Solarz AL, editors. Investing in children, youth, families, and communities: strengths-based research and policy. Washington, DC: American Psychological Association (2004). 233–50.

[19] Tatum B. “Why are all the black kids sitting together in the cafeteria?” And other conversations about race. New York, NY: Basic Books (1997). 10.1126/science.abn0865 34822268

[20] Rivas-Drake D Hughes D Way N. A preliminary analysis of associations among ethnic–racial socialization, ethnic discrimination, and ethnic identity among urban sixth graders. J Res Adolesc. (2009) 19:558–84. doi: 10.1111/j.1532-7795.2009.00607.x

[21] Felix ED You S. Peer victimization within the ethnic context of high school. J Community Psychol. (2011) 39:860–75. doi: 10.1002/jcop.20465

[22] Verkuyten M Jochem T. Ethnic discrimination and global self-worth in early adolescents: the mediating role of ethnic self-esteem. Int J Behav Dev. (2006) 30:107–16. doi: 10.1177/0165025406063573

[23] Verkuyten M Thijs J. Racist victimization among children in the Netherlands: the effect of ethnic group and school. Ethn Racial Stud. (2002) 25:310–31. doi: 10.1080/01419870120109502

[24] Seaton EK Caldwell CH Sellers RM Jackson JS. The prevalence of perceived discrimination among African American and Caribbean black youth. Dev Psychol. (2008) 44:1288–97. doi: 10.1037/a0012747, PMID: 18793063 PMC2556985

[25] Fisher CB Wallace SA Fenton RE. Discrimination distress during adolescence. J Youth Adolesc. (2000) 29:679–95. doi: 10.1023/A:1026455906512

[26] Skiba RJ Peterson RL Williams T. Office referrals and suspension: disciplinary intervention. Educ Treat Chil. (1997) 20:295–316.

[27] Townsend BL. The disproportionate discipline of African American learners: reducing school suspensions and expulsions. Except Child. (2000) 66:381–91. doi: 10.1177/001440290006600308

[28] Tenenbaum HR Ruck MD. Are teachers’ expectations different for racial minority than for NHW American students? A meta-analysis. J Educ Psychol. (2007) 99:253–73. doi: 10.1037/0022-0663.99.2.253

[29] Jia Y Way N Ling G Yoshikawa H Chen X Hughes D. The influence of student perceptions of school climate on socioemotional and academic adjustment: a comparison of Chinese and American adolescents. Child Dev. (2009) 80:1514–30. doi: 10.1111/j.1467-8624.2009.01348.x, PMID: 19765015

[30] Wentzel KR. Relations of social goal pursuit to social acceptance, classroom behavior, and perceived social support. J Educ Psychol. (1994) 86:173–82. doi: 10.1037/0022-0663.86.2.173

[31] McMahon SD Watts RJ. Ethnic identity in urban African American youth: exploring the links with self-worth, aggression, and other psychosocial variables. J Community Psychol. (2002) 30:411–31. doi: 10.1002/jcop.10013

[32] Romero AJ Roberts RE. The impact of multiple dimensions of ethnic identity on discrimination and adolescents’ self-esteem. J Appl Soc Psychol. (2003) 33:2288–305. doi: 10.1111/j.1559-1816.2003.tb01885.x

[33] Hoglund WL Hosan NE. The context of ethnicity peer victimization and adjustment problems in early adolescence. J Early Adolesc. (2013) 33:585–609. doi: 10.1177/0272431612451925

[34] Leadbeater BJ Hoglund WLG. The effects of peer victimization and physical aggression on changes in internalizing from first to third grade. Child Dev. (2009) 80:843–59. doi: 10.1111/j.1467-8624.2009.01301.x, PMID: 19489907

[35] Hoglund WL Leadbeater BJ. Managing threat: do social-cognitive processes mediate the link between peer victimization and adjustment problems in early adolescence? J Res Adolesc. (2007) 17:525–40. doi: 10.1111/j.1532-7795.2007.00533.x

[36] Colarossi LG Eccles JS. Differential effects of support providers on adolescents' mental health. Soc Work Res. (2003) 27:19–30. doi: 10.1093/swr/27.1.19

[37] Roeser R Eccles J Sameroff A. School as a context of early adolescents’ social–emotional development: a summary of research findings. Elem Sch J. (2000) 100:443–71. doi: 10.1086/499650

[38] Way N Reddy R Rhodes J. Students’ perceptions of school climate during the middle school years: association with trajectories of psychological and behavioral adjustment. Am J Community Psychol. (2007) 40:194–213. doi: 10.1007/s10464-007-9143-y 17968655

[39] Lusk E. M. Taylor M. J. Nanney J. T., & Austin C. C. (2010). Biracial identity and its relation to self-esteem and depression in mixed black/white biracial individuals. J Ethnic Cult Divers Soc Work 19, 109–126. doi: 10.1080/15313201003771783

[40] Choi Y Harachi TW Gillmore MR Catalano RF. Are multicultural adolescents at greater risk? Comparisons of rates, patterns, and correlates of substance use and violence between monoracial and multiracial adolescents. Am J Orthopsychiatry. (2006) 76:86–97. doi: 10.1037/0002-9432.76.1.86, PMID: 16569131 PMC3292211

[41] Jackson KF Lecroy CW. The influence of race and ethnicity on substance use and negative activity involvement among monoracial and multiracial adolescents of the southwest. J Drug Educ. (2009) 39:195–210. doi: 10.2190/DE.39.2.f, PMID: 19999705

[42] Udry JR Li RM Hendrickson-Smith J. Health and behavior risks of adolescents with mixed-race identity. Am J Public Health. (2003) 93:1865–70. doi: 10.2105/AJPH.93.11.1865, PMID: 14600054 PMC1448064

[43] Chavez GF Sanchez DT. A clearer picture of multiracial substance use: rates and correlates of alcohol and tobacco use in multiracial adolescents and adults. Race Soc Probl. (2010) 2:1–18. doi: 10.1007/s12552-010-9023-1

[44] Shih M Sanchez DT. Perspectives and research on the positive and negative implications of having multiple racial identities. Psychol Bull. (2005) 131:569–91. doi: 10.1037/0033-2909.131.4.569 16060803

[45] Crawford SE Alaggia R. The best of both worlds? Family influences on mixed race youth identity development. Qual Soc Work. (2008) 7:81–98. doi: 10.1177/1473325007086417

[46] Terry RL Winston CE. Personality characteristic adaptations: multiracial adolescents’ patterns of racial self-identification change. J Res Adolesc. (2010) 20:432–55. doi: 10.1111/j.1532-7795.2010.00638.x

[47] Albuja AF Sanchez DT Gaither SE. Identity denied: comparing American or white identity denial and psychological health outcomes among bicultural and biracial people. Personal Soc Psychol Bull. (2019) 45:416–30. doi: 10.1177/01467218788553, PMID: 30084303

[48] Davis LL Broome ME Cox RP. Maximizing retention in community-based clinical trials. J Nurs Scholarsh. (2002) 34:47–53. doi: 10.1111/j.1547-5069.2002.00047.x 11901967

[49] Child and Family Center. CFC youth questionnaire. Eugene, OR: Child and Family Center (2001).

[50] Metzler CW Biglan A Rusby JC Sprague JR. Evaluation of a comprehensive behavior management program to improve school-wide positive behavior support. Educ Treat Child. (2001) 24:448–79.

[51] Phinney JS. The multigroup ethnic identity measure: a new scale for use with diverse groups. J Adolesc Res. (1992) 7:156–76. doi: 10.1177/074355489272003

[52] Dishion TJ Kavanagh K. Intervening in adolescent problem behavior: a family-centered approach. New York, NY: Guilford (2003).

[53] Chang H Shaw DS Shelleby EC Dishion TJ Wilson MN. The long-term effectiveness of the family check-up on peer preference: parent-child interaction and child effortful control as sequential mediators. J Abnorm Child Psychol. (2017) 45:705–17. doi: 10.1007/s10802-016-0198-9, PMID: 27558394 PMC5927574

[54] Daniels M Wang C Marcus B. Fully Bayesian inference under ignorable missingness in the presence of auxiliary covariates. Bioemtrics. (2014) 70:62–72. doi: 10.1111/biom.12121, PMID: 24571539 PMC4007313

[55] Cai JH Song XY Hser YI. A Bayesian analysis of mixture structural equation models with non-ignorable missing responses and covariates. Stat Med. (2010) 29:1861–74. doi: 10.1002/sim.3915, PMID: 20680980

[56] Hu L Bentler PM. Cutoff criteria for fit indexes in covariance structure analyses: conventional criteria versus new alternatives. Struct Equ Model. (1999) 6:1–55. doi: 10.1080/10705519909540118

[57] Awang Z. Research methodology and data analysis. 2nd ed. Universiti Teknologi Mara, Singapore: UiTM Press (2012).

[58] Kline RB. Principles and practice of structural equation modeling. New York, NY: The Guilford Press (2005).

[59] Roberts RE Phinney JS Masse LC Chen YR Roberts CR Romero A. The structure of ethnic identity of young adolescents from diverse ethnocultural groups. J Early Adolesc. (1999) 19:301–22. doi: 10.1177/02724316990190030

[60] Brown CS Alabi BO Huynh VW Masten CL. Ethnicity and gender in late childhood and early adolescence: group identity and awareness of bias. Dev Psychol. (2011) 47:463–71. doi: 10.1037/a0021819 21219069

[61] Hughes D Witherspoon DP Rivas-Drake D West-Bey ND. Received ethnic–racial socialization messages and youth’ academic and behavioral outcomes: examining the mediating role of ethnic identity and self-esteem. Cultur Divers Ethnic Minor Psychol. (2009) 15:112–24. doi: 10.1037/a0015509, PMID: 19364198

[62] Fisher S Reynolds JL Hsu W Barnes J Tyler K. Examining multiracial youth in context: ethnic identity development and mental health outcomes. J Youth Adolesc. (2014) 43:1688–99. doi: 10.1007/s10964-014-0163-2, PMID: 25100614

[63] Quillian L Redd R. The friendship networks of multiracial adolescents. Soc Sci Res. (2009) 38:279–95. doi: 10.1016/j.ssresearch.2008.09.002, PMID: 19827177 PMC6258020

[64] Sugai G Horner RH. Introduction to the special series on positive behavior support in schools. J Emot Behav Disord. (2002) 10:130–5. doi: 10.1177/10634266020100030101

